# Outcomes in biomarker-selected subgroups from the KESTREL study of durvalumab and tremelimumab in recurrent or metastatic head and neck squamous cell carcinoma

**DOI:** 10.1007/s00262-024-03643-3

**Published:** 2024-03-02

**Authors:** Tanguy Y. Seiwert, Sophie Wildsmith, Jérôme Fayette, Kevin Harrington, Maura Gillison, Myung-Ju Ahn, Shunji Takahashi, Jared Weiss, Jean-Pascal Machiels, Shrujal Baxi, Valerie Baker, Brent Evans, Nassim Morsli, Jill Walker, Katia Real, Anne L’Hernault, Amanda Psyrri

**Affiliations:** 1 Department of Oncology, Sidney Kimmel Comprehensive Cancer Center, Johns Hopkins University, Baltimore, MD USA; 2 Oncology R&D, AstraZeneca, Cambridge, UK; 3 Centre de Lutte Contre le Cancer Léon Bérard, Lyon-I University, Lyon, France; 4 Division of Radiotherapy and Imaging, The Royal Marsden/The Institute of Cancer Research NIHR Biomedical Research Centre, London, UK; 5 Department of Thoracic Head and Neck Medical Oncology, University of Texas MD Anderson Cancer Center, Houston, TX USA; 6 Division of Hematology-Oncology, Sungkyunkwan University School of Medicine, Samsung Medical Center, Seoul, South Korea; 7 Department of Medical Oncology, The Cancer Institute Hospital of the Japanese Foundation for Cancer Research, Tokyo, Japan; 8 Lineberger Comprehensive Cancer Center, University of North Carolina, Chapel Hill, NC USA; 9 Department of Medical Oncology, Institut Roi Albert II, Cliniques Universitaires Saint-Luc and Institute for Experimental and Clinical Research (IREC, pôle MIRO), Université Catholique de Louvain (UCLouvain), Brussels, Belgium; 10 Department of Medicine, Memorial Sloan Kettering Cancer Center, New York, NY USA; 11 Statistics, AstraZeneca, Gaithersburg, MD USA; 12 Department of Internal Medicine, Section of Medical Oncology, Attikon University Hospital, National Kapodistrian University of Athens, Athens, Greece

**Keywords:** Immune checkpoint inhibitors, Head and neck neoplasms, Biomarkers, Programmed cell death-1 receptor, Immunotherapy

## Abstract

**Background:**

Selective biomarkers may improve outcomes in patients with recurrent or metastatic head and neck squamous cell carcinoma (R/M HNSCC) treated with immune checkpoint inhibitor therapy. We investigated three independent biomarkers for association with efficacy in the randomized, phase III KESTREL study (NCT02551159) of first-line durvalumab monotherapy or durvalumab plus tremelimumab versus the EXTREME regimen: programmed cell death ligand-1 (PD-L1) immunohistochemistry, blood tumor mutational burden (bTMB) via circulating tumor DNA, and neutrophil-to-lymphocyte ratio (NLR).

**Methods:**

Tumor or blood samples from patients enrolled in the KESTREL study were analyzed for PD-L1, bTMB, and NLR. Associations with overall survival (OS) or objective response rates (ORRs) were evaluated based on prespecified cut-offs for PD-L1 (tumor cell [TC] ≥ 50%/immune cell ≥ 25% or TC ≥ 25%), bTMB (≥ 16 mutations [mut] per megabase [Mb]), and NLR (≤ 7). Ad hoc analyses of exploratory cut-offs were performed.

**Results:**

Prespecified or exploratory cut-offs for PD-L1 did not enrich for ORR or OS for durvalumab monotherapy or durvalumab plus tremelimumab versus EXTREME. In the bTMB ≥ 16 mut/Mb subgroup, OS hazard ratios (95% confidence interval) for durvalumab monotherapy and durvalumab plus tremelimumab versus EXTREME were 0.90 (0.48–1.72) and 0.69 (0.39–1.25), respectively. Complete response rates were 8.6% with durvalumab plus tremelimumab and 4.3% with EXTREME (≥ 16 mut/Mb subgroup). No improvement in OS was observed for durvalumab monotherapy or durvalumab plus tremelimumab versus EXTREME at prespecified or exploratory NLR cut-offs.

**Conclusions:**

bTMB demonstrated potential utility for selecting patients with R/M HNSCC who benefited from durvalumab with or without tremelimumab versus EXTREME.

*Trial registration* ClinicalTrials.gov identifier NCT02551159.

**Supplementary Information:**

The online version contains supplementary material available at 10.1007/s00262-024-03643-3.

## Introduction

Recurrent or metastatic head and neck squamous cell carcinoma (R/M HNSCC) exhibits clinical and molecular heterogeneity and is associated with poor prognosis [1, 2]. High genomic instability and intratumoral genetic heterogeneity of HNSCC may explain treatment resistance and the tendency for locoregional recurrence in some patients [3, 4]. Moreover, survival outcomes in patients with HNSCC vary based on primary tumor location and human papillomavirus (HPV) status [1, 4, 5]. Immune checkpoint inhibitors (ICIs) are a treatment option for patients with R/M HNSCC; but only a subset of patients benefit from first-line treatment with ICIs [6, 7], as observed across many cancers [8]. Validated biomarkers that identify patients who are most likely to respond to ICIs may improve patient outcomes and avoid unnecessary toxicity and costs.

Several promising biomarkers, including programmed cell death ligand-1 (PD-L1), blood tumor mutational burden (bTMB) via circulating tumor DNA, and neutrophil-to-lymphocyte ratio (NLR), were identified in the EAGLE study (NCT02369874) of durvalumab with or without tremelimumab versus chemotherapy in patients with R/M HNSCC who progressed following definitive therapy [9, 10]. PD-L1 was further evaluated as a biomarker in the KESTREL study (NCT02551159) of durvalumab with or without tremelimumab versus the EXTREME regimen in patients with R/M HNSCC who had not received prior systemic therapy [11]. The primary objective of the KESTREL study was to assess the overall survival (OS) of durvalumab monotherapy versus the EXTREME regimen in patients with R/M HNSCC whose tumors expressed high levels of PD-L1 (PD-L1 tumor cell [TC] ≥ 50%/immune cell [IC] ≥ 25%) [11]. Secondary objectives included assessment of the efficacy of durvalumab monotherapy versus the EXTREME regimen in all randomized patients, and assessment of the efficacy of durvalumab plus tremelimumab combination therapy versus the EXTREME regimen in patients with PD-L1-high expression and in all randomized patients [11].

PD-L1 is the most widely used biomarker for selecting patients for ICI therapy in clinical practice and has been evaluated in phase III studies of patients with HNSCC [7, 10, 12, 13]. Long-term benefit was demonstrated for patients with platinum-refractory R/M HNSCC treated with nivolumab, irrespective of PD-L1 status, in the CheckMate 141 study (NCT02105636) [13]. In contrast, in the KEYNOTE-048 study (NCT02358031), survival benefit with first-line pembrolizumab versus chemotherapy was limited to patients with PD-L1-high expression (combined positive score [CPS] ≥ 20) [7, 14]. In the EAGLE study, median OS was longer for durvalumab monotherapy versus chemotherapy in patients with TC expression ≥ 25% versus < 25% [10]. However, unexpectedly, OS was also longer for durvalumab monotherapy versus chemotherapy in a small population of patients with TC expression < 1% [10]. Of 823 patients randomized in the KESTREL study (durvalumab monotherapy, n = 204; durvalumab plus tremelimumab, n = 413; EXTREME regimen, n = 206), 46.5% (n = 383) had tumors with PD-L1-high expression (PD-L1 TC ≥ 50%/IC ≥ 25%) [11]. The primary endpoint of the KESTREL study was not met; in patients with PD-L1-high expression, OS was comparable between durvalumab monotherapy and the EXTREME regimen (median OS, 10.9 vs. 10.9 months, respectively; hazard ratio [HR] = 0.96) [11]. In addition, no difference in OS was observed between durvalumab plus tremelimumab and the EXTREME regimen in patients with PD-L1-high expression (median OS, 11.2 vs. 10.9 months, respectively; HR = 1.05) and in all randomized patients (median OS, 10.7 vs. 10.3 months, respectively; HR = 1.04) [11]. Whether an alternative PD-L1 scoring algorithm could predict efficacy outcomes in patients enrolled in the KESTREL study remained to be assessed.

TMB has emerged as a promising biomarker for ICIs. In the US, patients with unresectable or metastatic solid tumors with TMB ≥ 10 mutations per megabase (mt/Mb), who have progressed following prior treatment and without alternative treatment options, are eligible for pembrolizumab treatment based on the results of the KEYNOTE-158 study (NCT02628067) [15, 16]. In R/M HNSCC, the EAGLE study showed a significant OS benefit for durvalumab with or without tremelimumab versus chemotherapy for patients with bTMB ≥ 16 mut/Mb (HR = 0.39 [confidence interval (CI) 0.20–0.76] and 0.38 [95% CI 0.19–0.78], respectively) [9]. The utility of bTMB ≥ 16 mut/Mb for predicting survival in patients with R/M HNSCC treated with these therapies in the front-line setting had not previously been determined.

NLR is a standard clinical assessment of tumor-induced inflammation based on blood cell counts. Although generally regarded as a prognostic marker of survival in HNSCC [17], it has gained interest as a predictive marker for ICIs [18]. High baseline NLR had a strong inverse relationship with OS and progression-free survival (PFS) in patients receiving anti-PD-1 therapy for metastatic head and neck cancer, regardless of PD-L1 expression [19]. A recent study found that low baseline NLR was associated with improved response in patients with R/M HNSCC receiving ICIs, and that on-treatment high NLR (≥ 4) had a significant negative correlation with OS and PFS [20]. These findings were further supported by the EAGLE study, which showed a statistically significant OS benefit in patients with R/M HNSCC and an NLR ≤ 7 who were treated with durvalumab monotherapy versus chemotherapy (HR = 0.75, 95% CI 0.57–0.97) [9]. As NLR is a simple, inexpensive, and routine assessment, it is of interest to assess its utility to select patients with R/M HNSCC for ICI therapy.

Herein, we analyzed data from the KESTREL study to evaluate the predictive utility of validated, prespecified cut-offs of PD-L1 expression, bTMB, and NLR. We further evaluated exploratory cut-offs for the selection of patients using these biomarkers in ad hoc analyses.

## Methods

### Patients

Patients with evaluable samples for biomarker analysis from the KESTREL study were included. Eligible patients were aged ≥ 18 years with histologically or cytologically confirmed R/M HNSCC (oral cavity, oropharynx, hypopharynx, or larynx) not amenable to local, curative therapy with surgery or radiation. Patients were eligible if they had not received prior systemic therapy for R/M disease, unless it was given as part of multimodal treatment for locally advanced or recurrent disease, and recurrence had occurred > 6 months from the last platinum dose.

The KESTREL study was conducted in accordance with the Declaration of Helsinki and was consistent with International Conference on Harmonization and Good Clinical Practice guidelines, and applicable regulatory requirements. Written informed consent from participants was obtained before performing any protocol-related procedures and use of biological samples.

### Study design and treatment

The KESTREL study (NCT02551159) was a randomized, open-label, multicenter, global phase III study. The study was conducted at 197 sites in 23 countries. Patients were randomized 2:1:1 to durvalumab plus tremelimumab (concurrent durvalumab 1500 mg every 4 weeks [Q4W] and tremelimumab 75 mg Q4W for a maximum of 4 doses), durvalumab monotherapy (1500 mg Q4W), or the EXTREME regimen (cisplatin 100 mg/m^2^ of body surface area or carboplatin at an area under the curve of 5 mg/mL/min on Day 1, at the discretion of the investigator, and 5-fluorouracil 1000 mg/m^2^/day on Days 1 through 4 of every 3-week cycle, as well as cetuximab 400 mg/m^2^ on Day 1, followed by 250 mg/m^2^ every week). Randomization was stratified by tumor location (oropharyngeal or non-oropharyngeal), smoking history (> 10 or ≤ 10 pack-years), and PD-L1 status (positive or negative) at cut-off of TC ≥ 25%. Further stratification by HPV status (positive or negative) was performed for patients with oropharyngeal cancer.

### Study objectives

Assessment of the efficacy of durvalumab monotherapy and durvalumab plus tremelimumab versus the EXTREME regimen in patients with PD-L1 TC ≥ 50%/IC ≥ 25% and in all randomized patients, were primary and secondary objectives of the KESTREL study [11]. In addition to the prespecified cut-off of TC ≥ 50%/IC ≥ 25% and stratification cut-off of TC ≥ 25%, an ad hoc analysis was performed to assess efficacy at exploratory cut-offs of PD-L1. Exploratory cut-offs were selected based on published reports for ICI treatment in R/M HNSCC [7, 12–14, 21].

A secondary objective of the KESTREL study was to assess efficacy in patients selected for high bTMB level at a prespecified cut-off of ≥ 16 mut/Mb. Efficacy was assessed in terms of OS and objective response rate (ORR), as defined previously [11]. In an ad hoc analysis, OS was also assessed at exploratory cut-offs of bTMB ≥ 8, ≥ 10, ≥ 12, ≥ 14, ≥ 18, and ≥ 20 mut/Mb to confirm the optimal cut-off.

Assessment of OS in patients selected for low NLR at a cut-off of ≤ 7 was a prespecified exploratory objective of the KESTREL study. An ad hoc analysis assessed OS in patients with baseline NLR ≤ 4 or ≤ 8, selected based on published reports for ICI treatment in R/M HNSCC [20].

### Biomarker assessments

PD-L1 expression was determined using formalin-fixed, paraffin-embedded tumor tissue obtained from recently acquired (preferred) or archival samples (≤ 3 years before screening). PD-L1 testing was performed using the VENTANA PD-L1 (SP263) Assay (Roche Tissue Diagnostics, Tucson, AZ, USA) in a College of American Pathologists-accredited and Clinical Laboratory Improvement Amendments-certified central laboratory. Pathologists were trained by the manufacturer for validated scoring at the stratification cut-off of TC ≥ 25% and the primary endpoint population of TC ≥ 50%/IC ≥ 25%.

bTMB level was determined from baseline blood samples. Sampling ceased after a protocol amendment and, thus, was not continued throughout the duration of the study. Blood was collected in K2-EDTA tubes, processed for plasma collection, and stored at − 80 °C. bTMB was assessed using the GuardantOMNI assay, as previously described [22–24].

Absolute neutrophil count and absolute lymphocyte count were assessed according to local standards to derive NLRs.

### bTMB and variant analysis

Cell-free DNA next-generation sequencing analysis was performed at Guardant Health, Inc. (Redwood City, CA, USA). The 2.145 Mb GuardantOMNI assay identifies single nucleotide variants (SNVs) and indels in 496 genes, copy number amplifications (106 genes), fusions (21 genes), microsatellite instability (MSI)-high status, and TMB [24, 25]. bTMB was reported as mut/Mb by the GuardantOMNI algorithm, which includes all somatic synonymous and non-synonymous SNVs and indels, excluding germline, clonal hematopoiesis of indeterminate potential, driver, and resistance mutations, with statistical adjustment for sample-specific tumor shedding and molecular coverage. Samples with low tumor shedding (all somatic mutations < 0.3% maximum somatic allele fraction) or low unique molecule coverage were identified as bTMB-unevaluable. Validation of bTMB has been previously described [23, 26].

### Statistical analyses

Median OS values and 95% CIs were computed using the Kaplan–Meier method. OS HRs and 95% CIs were estimated using a stratified Cox proportional hazards model; ties were handled using the Efron approach, and 95% CIs were calculated using a profile likelihood approach. ORRs were calculated as the percent of patients with partial or complete responses of the total subgroup; 95% CIs were computed using exact binomial distribution. Statistical hypothesis testing for key secondary endpoints commenced only if the primary endpoint reached statistical significance. As the primary objective of the KESTREL study was not met [11], all statistics reported are descriptive.

## Results

### PD-L1

PD-L1 was evaluable in tumor samples from 820/823 (99.6%) of all randomized patients in the KESTREL study (Supplementary Table 1). PD-L1 prevalence was 31.1% (n = 256/823) at the TC ≥ 25% cut-off, consistent with the EAGLE study [10], and 46.7% (n = 383/820) at the TC ≥ 50%/IC ≥ 25% cut-off (subsubgroup for primary endpoint analysis; Supplementary Table 1). For the PD-L1 analysis, 51% of samples were recently acquired (< 3 months old) and 49% were archival.

Data from the primary analysis of the KESTREL study have previously been reported [11]. In the primary analysis, median OS was similar in patients with PD-L1 TC ≥ 50%/IC ≥ 25% in all treatment arms, and there was no significant OS benefit with durvalumab monotherapy versus the EXTREME regimen in this subgroup [11]. However, median OS and OS rates at 12, 18, and 24 months were numerically higher in patients treated with durvalumab monotherapy or durvalumab plus tremelimumab with TC ≥ 50%/IC ≥ 25% versus those with TC < 50% and IC < 25% (Supplementary Table 2) [11]. ORRs and complete response rates (CRRs) were similar in the TC ≥ 50%/IC ≥ 25% subgroup versus all randomized patients for durvalumab monotherapy (16.2% vs. 17.2% and 0 vs. 1.5%, respectively) and the EXTREME regimen (50.0% vs. 49.0% and 3.2% vs. 1.9%, respectively) [11]. The ORRs and CRRs were numerically higher in the TC ≥ 50%/IC ≥ 25% subgroup versus all randomized patients for durvalumab plus tremelimumab (25.3% vs. 21.8% and 5.3% vs. 3.9%, respectively) [11].

**Fig. 1:**
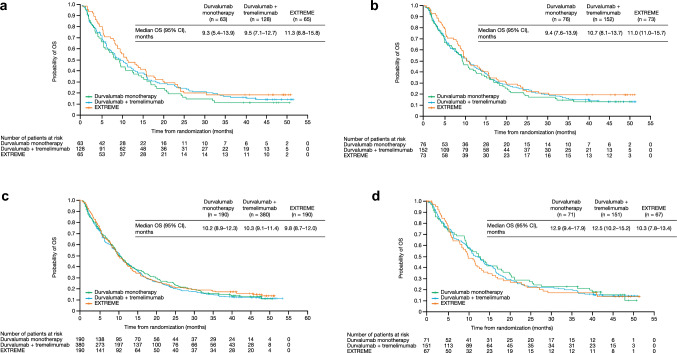
Analysis of OS using different PD-L1 cut-offs. **a** TC ≥ 25%, **b** CPS ≥ 20, **c** CPS ≥ 1, and **d** IC ≥ 25%. CI, confidence interval; CPS, combined positive score; IC, immune cell; OS, overall survival; PD-L1, programmed cell death ligand-1; TC, tumor cell

Median OS in patients with TC ≥ 25%, CPS ≥ 1, CPS ≥ 20, or IC ≥ 25% was similar across treatment arms (Fig. 1a–d). OS HRs across most TC or IC cut-offs showed no benefit for durvalumab monotherapy or durvalumab plus tremelimumab versus the EXTREME regimen (Supplementary Fig. 1). Median OS was generally similar in patients with TC < 25%, CPS < 20, or IC < 25% who were treated with durvalumab monotherapy, durvalumab plus tremelimumab, or the EXTREME regimen (data not shown). Median OS was longer for durvalumab plus tremelimumab and shorter for durvalumab monotherapy versus the EXTREME regimen in patients with CPS < 1 (data not shown).

In an ad hoc analysis of ORR at additional PD-L1 cut-offs, IC ≥ 50% showed the most enrichment, although this subgroup was small (Supplementary Table 3). In the durvalumab monotherapy arm, the highest ORR was observed in patients with IC ≥ 50% (23.8%; n = 5/21). At the cut-off of IC ≥ 50% in the durvalumab plus tremelimumab arm, 35.7% (n = 10/28) of patients had an objective response and 17.9% (n = 5/28) of patients had a complete response, whereas in the EXTREME regimen, 30.0% (n = 3/10) of patients had an objective response and no patients had a complete response. In the durvalumab plus tremelimumab arm, there appeared to be an overall numerical trend in increasing response rate with increasing TC or IC cut-offs (Supplementary Table 3).

### bTMB

Blood samples for biomarker analysis were available for 73% (n = 598/823) of randomized patients. Variant landscape analysis in processed samples (n = 536) was reflective of HNSCC, with a high prevalence of *TP53* (70%), *PIK3CA* (29%), *KMT2D* (26%), *NOTCH1* (18%), *FAT1* (18%), and *LRP1B* (18%) alterations (Supplementary Fig. 2). bTMB was determined for 56% (n = 461/823) of randomized patients, comprising the bTMB evaluable population (BEP; Supplementary Fig. 3). The prevalence of bTMB ≥ 16 mut/Mb was 26.0% (n = 120/461) in the BEP.

Baseline demographics and disease characteristics in the BEP were similar to all randomized patients [11], although some differences were apparent between the bTMB ≥ 16 mut/Mb and bTMB < 16 mut/Mb subgroups (Supplementary Table 4). The bTMB ≥ 16 mut/Mb subgroup was enriched for patients who had PD-L1 expression TC ≥ 25% or poorer performance status, and included fewer patients with oral cavity tumors, more patients with laryngeal tumors, and more patients with metastatic versus recurrent disease, compared with the bTMB < 16 mut/Mb subgroup (Supplementary Table 4).

**Fig. 2:**
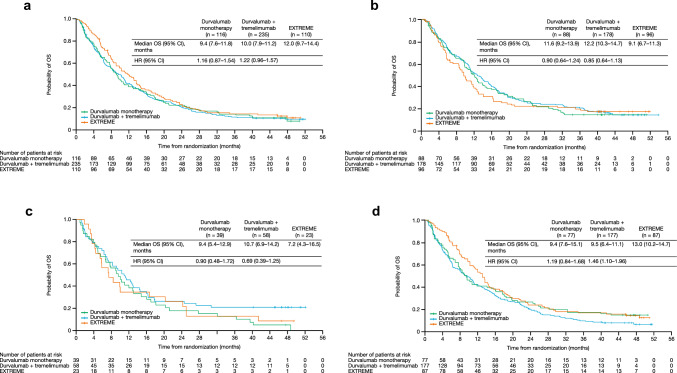
Analysis of OS in patient subgroups categorized by bTMB status. **a** Patients with bTMB evaluable samples, **b** bTMB unknown, **c** bTMB ≥ 16 mut/Mb, and **d** bTMB < 16 mut/Mb. bTMB, blood tumor mutation burden; CI, confidence interval; HR, hazard ratio; mut/Mb, mutations per megabase; OS, overall survival

OS in the BEP was not consistent with the OS for all randomized patients (Fig. 2a) [11]. In patients treated with the EXTREME regimen, the median OS was 12.0 months in the BEP compared with 10.3 months in all randomized patients [11] and 9.1 months in patients with unknown bTMB results (Fig. 2b). Therefore, it is more appropriate to compare OS in the bTMB-selected populations comprising the BEP than to all randomized patients. For durvalumab monotherapy versus the EXTREME regimen, the OS HR was 0.90 (95% CI 0.48–1.72) in the bTMB ≥ 16 mut/Mb subgroup (Fig. 2c), compared with 1.16 (95% CI 0.87–1.54) in the BEP subgroup (Fig. 2a). The OS HR for durvalumab plus tremelimumab versus EXTREME was 0.69 (95% CI 0.39–1.25) in the bTMB ≥ 16 mut/Mb subgroup (Fig. 2c), compared with 1.22 (95% CI 0.96–1.57) in the BEP subgroup (Fig. 2a). In patients treated with the EXTREME regimen, OS was poorer in the bTMB ≥ 16 mut/Mb (median OS 7.2, 95% CI 4.3–16.5) versus the bTMB < 16 mut/Mb (median OS 13.0, 95% CI 10.2–14.7) subgroup (Fig. 2c, d). In a subgroup analysis of OS by demographic characteristics in patients with bTMB ≥ 16 mut/Mb, OS HRs generally favored durvalumab plus tremelimumab versus the EXTREME regimen, although patient numbers were low, 95% CIs were wide, and differences were not statistically significant (Supplementary Fig. 4). In an ad hoc analysis of increasing cut-offs of bTMB, OS HRs for durvalumab monotherapy or durvalumab plus tremelimumab versus the EXTREME regimen were lower for bTMB ≥ 16 mut/Mb, compared with other bTMB cut-offs, although 95% CIs were wide and overlapping (Supplementary Fig. 5).

Higher ORRs were observed for durvalumab plus tremelimumab, but not with durvalumab monotherapy or the EXTREME regimen in the bTMB ≥ 16 mut/Mb subgroup, compared with the bTMB < 16 mut/Mb subgroup (Table 1). In the durvalumab monotherapy arm, ORR was 10.3% (95% CI 4.3–27.6) in the bTMB ≥ 16 mut/Mb subgroup and 18.2% (95% CI 11.1–28.4) in the bTMB < 16 mut/Mb subgroup. In the durvalumab plus tremelimumab arm, ORR was 31.0% (95% CI 21.6–50.3) in the bTMB ≥ 16 mut/Mb subgroup and 16.9% (95% CI 12.1–23.2) in the bTMB < 16 mut/Mb subgroup. In the EXTREME arm, ORR was 43.5% (95% CI 24.5–69.4) in the bTMB ≥ 16 mut/Mb subgroup and 55.2 (95% CI 44.6–65.3) in the bTMB < 16 mut/Mb subgroup. The CRR was higher in patients with bTMB ≥ 16 mut/Mb treated with durvalumab plus tremelimumab (8.6%), compared with durvalumab monotherapy (0.0%) or EXTREME (4.3%).

**Table 1:** ORR in patient subgroups categorized by bTMB status

ORR, n/N (%) [95% CI]	Durvalumab monotherapy	Durvalumab plus tremelimumab	EXTREME
	(n = 204)	(n = 413)	(n = 206)
bTMB evaluable	18/116 (15.5)	48/235 (20.4)	58/110 (52.7)
bTMB ≥ 16 mut/Mb	4/39 (10.3) [4.3–27.6]	18/58 (31.0) [21.6–50.3]	10/23 (43.5) [24.5–69.4]
bTMB < 16 mut/Mb	14/77 (18.2) [11.1–28.4]	30/177 (16.9) [12.1–23.2]	48/87 (55.2) [44.6–65.3]
bTMB unknown	17/88 (19.3) [12.4–28.9]	42/178 (23.6) [17.9–30.4]	43/96 (44.8) [35.2–54.8]

*bTMB* blood tumor mutational burden, *CI* confidence interval, *mut/Mb* mutations per megabase, *ORR* objective response rate

### NLR

**Fig. 3:**
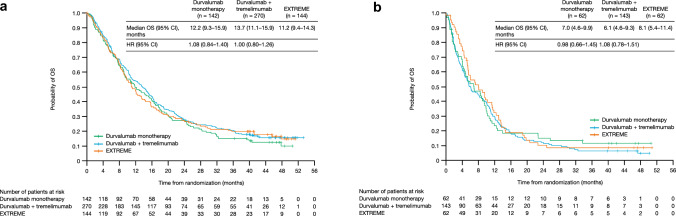
Analysis of OS in patient subgroups categorized by NLR status. Kaplan–Meier curves for the **a** NLR ≤ 7 and **b** NLR > 7 subgroups are shown. CI, confidence interval; HR, hazard ratio; NLR, neutrophil-to-lymphocyte ratio; OS, overall survival

NLR was evaluable for 100% (823/823) of all randomized patients, and the prevalence of NLR ≤ 7 was 68% (Supplementary Table 5). Median OS of patients with NLR ≤ 7 was longer than those with NLR > 7, irrespective of treatment arm (Fig. 3). In patients with NLR ≤ 7, OS HRs did not favor durvalumab monotherapy versus EXTREME (1.08 [95% 0.84–1.40]) or durvalumab plus tremelimumab versus EXTREME (1.00 [95% 0.80–1.26]).

In an ad hoc analysis, OS HR did not favor either ICI treatment arm in the NLR ≤ 4 subgroup (durvalumab monotherapy vs. EXTREME, 1.02 [95% CI 0.71–1.45]; durvalumab plus tremelimumab vs. EXTREME, 0.99 [95% CI 0.72–1.38]) or the NLR ≤ 8 subgroup (durvalumab monotherapy vs. EXTREME, 1.09 [95% CI 0.85–1.39]); durvalumab plus tremelimumab vs. EXTREME, 0.99 [95% CI 0.80–1.23]; Supplementary Table 6).

## Discussion

The KESTREL study did not meet its primary objective of improved OS in patients with PD-L1-high expression treated with durvalumab monotherapy versus the EXTREME regimen [11]. The CheckMate 651 study also failed to demonstrate significant OS benefit with nivolumab plus ipilimumab versus the EXTREME regimen in patients with PD-L1-high tumors (CPS ≥ 20), albeit with numerical OS improvement [27]. Only the KEYNOTE-048 study showed prolonged OS with pembrolizumab monotherapy versus the EXTREME regimen in patients selected for high PD-L1 expression (CPS ≥ 20) [7, 14]. The CheckMate 651 and KESTREL studies both had higher frequencies of subsequent immunotherapy use in the control arm than the KEYNOTE-048 study, suggesting that subsequent immunotherapy use may have confounded OS analysis [7, 11, 27]. Other factors that may have contributed to the difference in outcomes between the KESTREL, CheckMate 651, and KEYNOTE-048 studies, include differences in study design, eligibility criteria, regional variations, and the type of PD-L1 assay used [7, 11, 27]. For example, the KESTREL study used an SP263 antibody clone [11], whereas the KEYNOTE-048 study used a 22C3 antibody clone [7]. Previous findings have shown modest agreement of SP263 and 22C3 antibody clones using the CPS algorithm (75% overall percent agreement) [28].

In the EXTREME arm of the KESTREL study, patients who received subsequent immunotherapy (24.3%) had longer survival compared with those who did not, and the predictive value of PD-L1 for OS benefit was likely impacted by the effect of subsequent immunotherapy use [11]. This is further supported by our finding that median OS and OS rates at 12, 18, and 24 months were numerically higher in patients treated with durvalumab monotherapy or durvalumab plus tremelimumab with TC ≥ 50%/IC ≥ 25% versus those with TC < 50% and IC < 25%. In order to remove the influence of subsequent therapy, we considered response rates. Our results showed that patients were more likely to have a complete response when treated with durvalumab plus tremelimumab versus the EXTREME regimen, across PD-L1 subgroups; however, this was not the case for patients treated with durvalumab monotherapy. No substantial improvement in OS or ORR was seen when alternative cut-offs were used.

bTMB appeared to be effective in enriching for response to durvalumab plus tremelimumab, with an ORR of 31.0% in the bTMB ≥ 16 mut/Mb subgroup versus 16.9% in the bTMB < 16 mut/Mb subgroup. In the durvalumab plus tremelimumab arm, CRR was 8.6% in the bTMB ≥ 16 mut/Mb subgroup, which may have contributed to the flattened, long tail (plateau) of the OS Kaplan–Meier curve observed for this subgroup. Moreover, our finding that ORRs were improved in the bTMB ≥ 16 mut/Mb subgroup with durvalumab plus tremelimumab, but not with durvalumab monotherapy or the EXTREME regimen, indicates that bTMB may be predictive of response to durvalumab plus tremelimumab therapy in patients with bTMB ≥ 16 mut/Mb. Recent findings have also shown higher ORR in patients with advanced or metastatic solid tumors and bTMB ≥ 10 mut/Mb treated with ipilimumab plus nivolumab (22.5%) versus nivolumab alone (15.6%) [29].

In the EXTREME arm, OS was lower in the bTMB ≥ 16 mut/Mb subgroup (7.2 months) compared with the bTMB < 16 mut/Mb subgroup (13.0 months). These findings suggest that patients with a lower bTMB may be more likely to benefit from treatment with chemotherapy. When compared to the bTMB < 16 mut/Mb subgroup, more patients treated in the bTMB ≥ 16 mut/Mb subgroup had a poorer performance status, and were more likely to have metastatic than recurrent disease, suggesting that patients in the bTMB ≥ 16 mut/Mb subgroup may have had a poorer prognosis. Unfortunately, the bTMB analysis was impacted by low sample ascertainment and sub-optimal sample collection, such that bTMB results were only available for 56% of patients. These factors led to small bTMB ≥ 16 mut/Mb subgroups (n = 23; EXTREME regimen) with wide CIs. However, an ad hoc analysis of multiple cut-offs confirmed that bTMB ≥ 16 mut/Mb was optimal for this disease setting.

In the EAGLE study, bTMB was predictive of survival for durvalumab monotherapy and durvalumab plus tremelimumab [9]. These findings differ from the KESTREL study, where bTMB appeared to be most effective in the durvalumab plus tremelimumab arm. The utility of bTMB as a biomarker for this combination was previously observed in the MYSTIC non-small-cell lung cancer study, wherein a greater OS benefit over chemotherapy was observed in patients with bTMB ≥ 20 mut/Mb treated with durvalumab plus tremelimumab (HR = 0.49) than for durvalumab alone (HR = 0.72) [30]. Similarly, in the DANUBE metastatic urothelial carcinoma study, the OS benefit of patients with bTMB ≥ 24 mut/Mb treated with durvalumab and tremelimumab (HR = 0.56) was considerably improved versus durvalumab alone (HR = 1.02) [31].

Overall, there is evidence supporting the relationship between TMB and clinical activity of anti-programmed cell death-1 (PD-1)/PD-L1 antibodies as monotherapy and in combination with anti-cytotoxic T-lymphocyte-associated antigen 4 (CTLA-4) antibodies [32]. However, the clinical benefit of adding anti-CTLA-4 to anti-PD-1/PD-L1 therapy appears greatest in TMB-high tumors, which have increased tumor immunogenicity resulting from high expression of tumor neoantigens [32, 33]. It is possible that with a combination approach, anti-tumor immune responses are further enhanced by non-redundant targeting of the PD-1/PD-L1 and CTLA-4 signaling axes, or by specific tumor mutational features that benefit from CTLA-4 inhibition.

In contrast to previous observations from the EAGLE study [9], none of the NLR cut-offs assessed were predictive of OS benefit for durvalumab monotherapy or durvalumab plus tremelimumab versus the EXTREME regimen. However, subsequent therapy use may have confounded the survival results.

In summary, we investigated three independent selection methods to identify patients with R/M HNSCC most likely to benefit from treatment with durvalumab with and without tremelimumab. The results exemplify the imperfect nature of PD-L1 as a biomarker in HNSCC. bTMB showed promise as a biomarker of response and survival, particularly for the combination of durvalumab plus tremelimumab. This finding is consistent with our understanding of the mechanism of action of anti-PD-1/PD-L1 and anti-CTLA-4 antibodies, and may be useful in the future for directing these types of therapies.

### Supplementary Information

Below is the link to the electronic supplementary material.

Supplementary file1 (PDF 706 KB)

## Data Availability

Data underlying the findings described in this manuscript may be obtained in accordance with AstraZeneca’s data sharing policy described at https://astrazenecagrouptrials.pharmacm.com/ST/Submission/Disclosure. Data for studies directly listed on Vivli can be requested through Vivli at www.vivli.org. Data for studies not listed on Vivli could be requested through Vivli at https://vivli.org/members/enquiries-about-studies-not-listed-on-the-vivli-platform/. AstraZeneca Vivli member page is also available outlining further details: https://vivli.org/ourmember/astrazeneca/.

## Abbreviations

AJCC: American Joint Committee on Cancer
BEP: bTMB evaluable population
bTMB: Blood tumor mutational burden
CI: Confidence interval
CPS: Combined positive score
CRR: Complete response rate
CTLA-4: Cytotoxic T-lymphocyte-associated antigen 4
HNSCC: Head and neck squamous cell carcinoma
HPV: Human papillomavirus
HR: Hazard ratio
IC: Immune cell
ICI: Immune checkpoint inhibitor
MSI: Microsatellite instability
mut/Mb: Mutations per megabase
NLR: Neutrophil-to-lymphocyte ratio
ORR: Objective response rate
OS: Overall survival
PD-1: Programmed cell death-1
PD-L1: Programmed cell death ligand-1
PFS: Progression-free survival
Q4W: Every 4 weeks
R/M: Recurrent or metastatic
SNV: Single nucleotide variant
TC: Tumor cell
TMB: Tumor mutational burden
