# Supplementary material for: Outcomes in biomarker-selected subgroups from the KESTREL study of durvalumab and tremelimumab in recurrent or metastatic head and neck squamous cell carcinoma
Source: Cancer Immunol Immunother. 2024 Mar 2;73(4):70. doi: 10.1007/s00262-024-03643-3 (PMC10908636; doi:10.1007/s00262-024-03643-3)
Supplement: Supplementary file 1 — Supplementary file1 (PDF 706 KB) [file 262_2024_3643_MOESM1_ESM.pdf]

Supplementary Material

Cancer Immunology, Immunotherapy (submitted in 2023) - Tanguy Y. Seiwert et al.

Plain language summary

Why did we perform this research?

Head and neck squamous cell carcinomas (HNSCC) are a group of cancers that occur in the head and neck. HNSCC that comes back after treatment or that has spread from its original site is called recurrent or metastatic (R/M) HNSCC. Biomarkers (genes, proteins, or other substances that provide information about cancer) are being tested in patients with R/M HNSCC to understand if they can predict how well a patient responds to treatment. In the KESTREL study, patients with R/M HSNCC were treated with durvalumab with or without tremelimumab (both treatments that target the immune system to help the body fight cancer [immunotherapies]), or the EXTREME regimen (chemotherapy). The aim of this research was to find out if certain biomarkers could predict how well patients in the KESTREL study responded to treatment.

How did we perform this research?

Patients in the KESTREL study who had tissue or blood samples available for biomarker testing were included in this research. Patients' tissue or blood samples were tested for the following biomarkers: PD-L1 (a protein that stops the immune system from attacking cancer cells), blood tumor mutational burden (bTMB, a biomarker that measures the number of mutations within a tumor), and neutrophil-to-lymphocyte ratio (NLR, a biomarker that measures the level of different white blood cells within a persons' blood). The biomarkers were tested to see if they could predict how well a patient responded to treatment with durvalumab with or without tremelimumab or the EXTREME regimen.

What were the findings of this research?

PD-L1 and NLR biomarkers could not predict how long a patient lived after starting treatment (overall survival) or how many patients had a decrease in the size or number of their tumors (objective response rate) after treatment with durvalumab with or without tremelimumab or the EXTREME regimen. High levels of the bTMB biomarker (defined as ≥16 mutations per megabase of DNA) predicted when durvalumab with or without tremelimumab worked better than the EXTREME regimen. Patients who had high levels of the bTMB biomarker that were treated with durvalumab

with or without tremelimumab were more likely to live longer after starting treatment and were more likely to have a decrease in the size or number of their tumors compared with patients who had high levels of the bTMB biomarker that were treated with the EXTREME regimen.

What are the implications of this research?

bTMB was identified as a potential biomarker for selecting patients with R/M HNSCC who benefited from treatment with durvalumab with or without tremelimumab versus the EXTREME regimen.

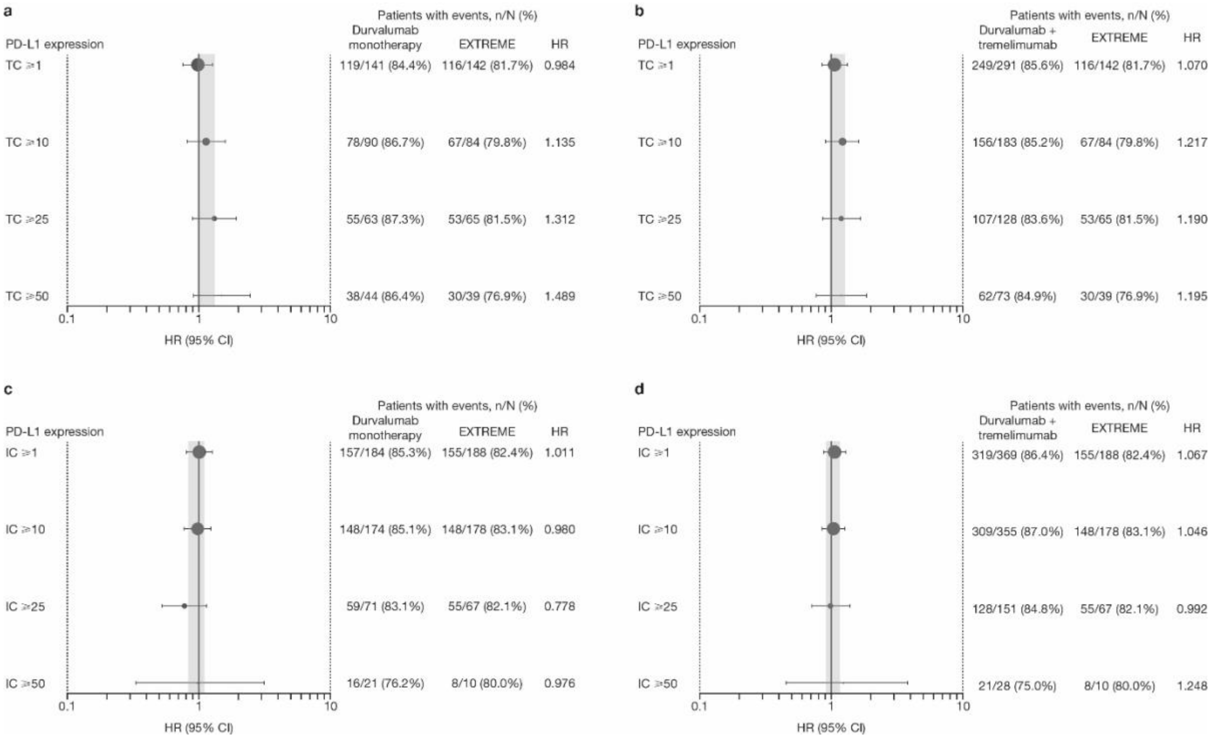

Supplementary Figure 1 OS HRs versus the EXTREME regimen for **a**, durvalumab monotherapy using TC PD-L1 cut-offs, **b**, durvalumab plus tremelimumab using TC PD-L1 cut-offs, **c**, durvalumab monotherapy using IC PD-L1 cut-offs, and **d**, durvalumab plus tremelimumab using IC PD-L1 cut-offs

CI, confidence interval; HR, hazard ratio; IC, immune cell; OS, overall survival; PD-L1, programmed cell death ligand-1; TC, tumor cell

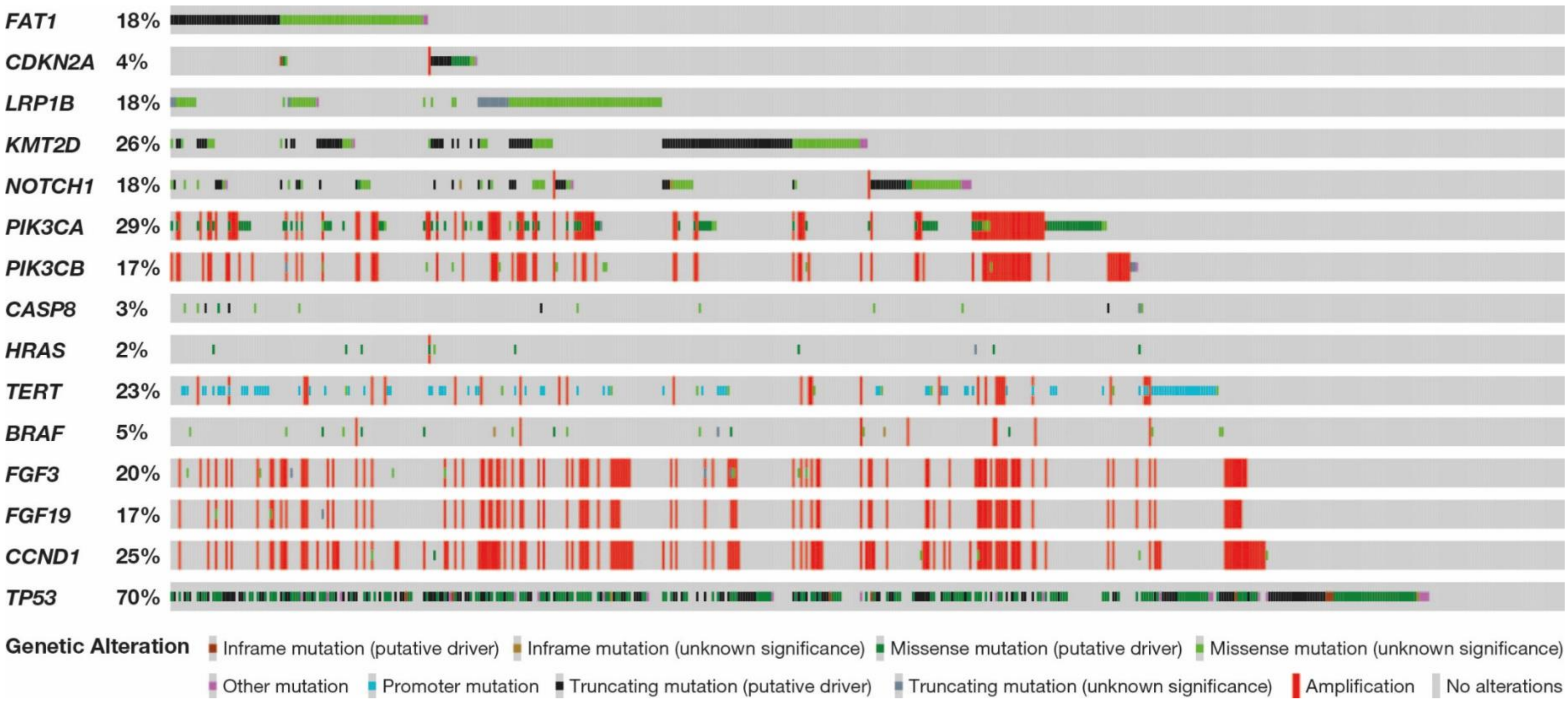

Supplementary Figure 2 Variant landscape analysis in processed blood samples (n=536)

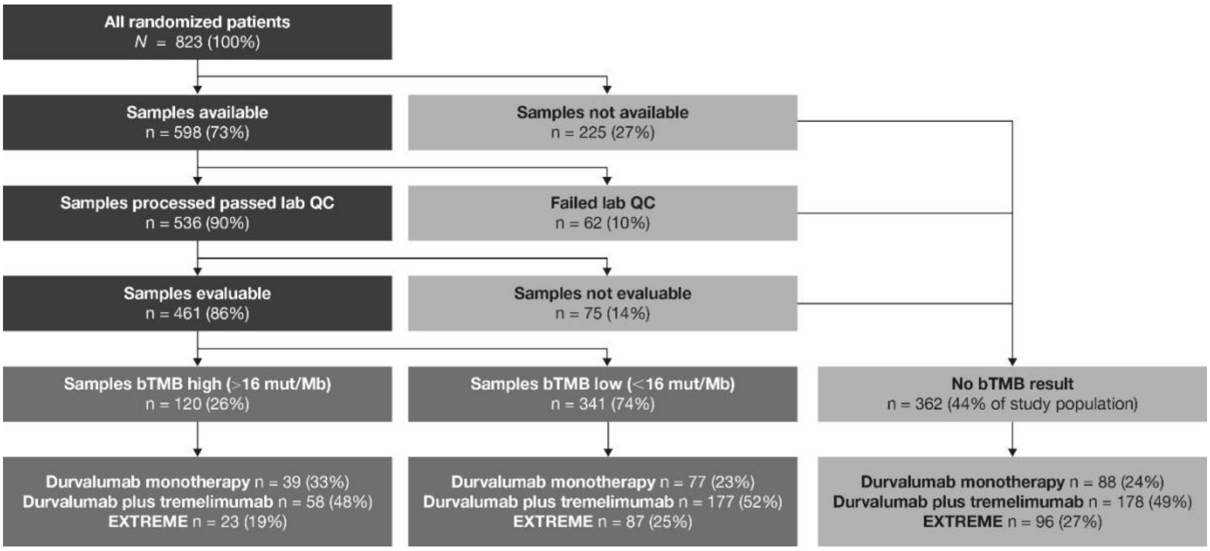

Supplementary Figure 3 Patient disposition for bTMB analysis

bTMB, blood tumor mutation burden; mut/Mb, mutations per megabase; QC, quality control

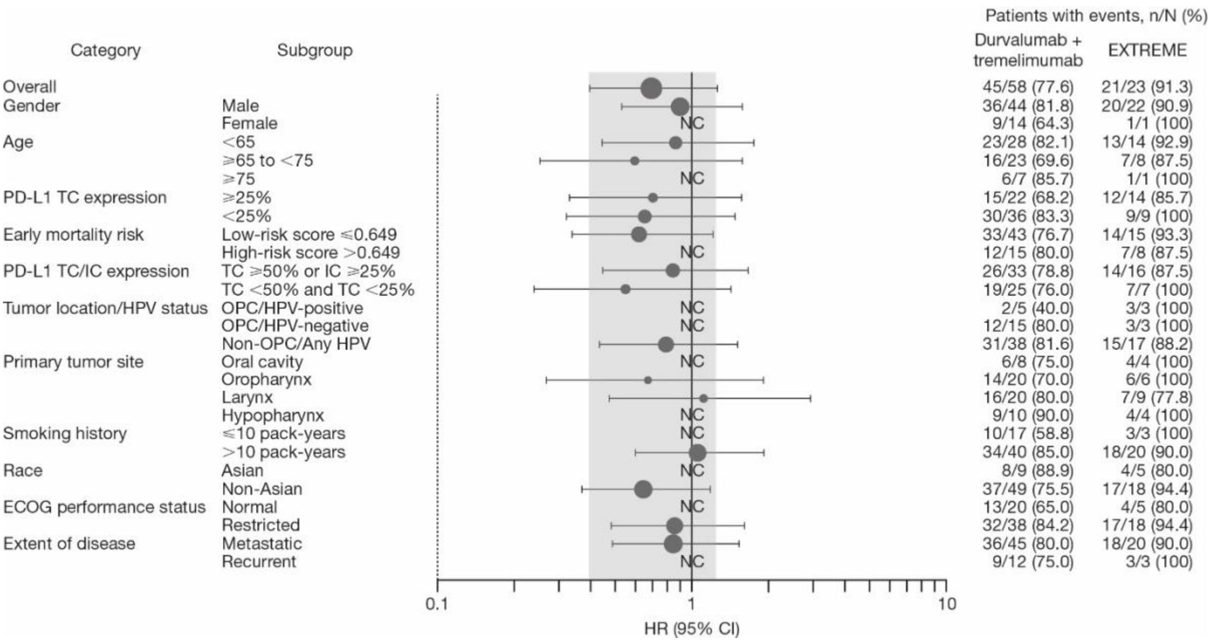

Supplementary Figure 4 OS HR versus the EXTREME regimen in the bTMB ≥16 mut/Mb subgroup, by demographic characteristics for durvalumab plus tremelimumab

bTMB, blood tumor mutation burden; CI, confidence interval; ECOG, Eastern Cooperative Oncology Group; HPV, human papillomavirus; HR, hazard ratio; IC, immune cell; mut/Mb, mutations per megabase; NC, not calculable; OPC, oropharyngeal cancer; OS, overall survival; PD-L1, programmed cell death ligand-1; PS, performance status; TC, tumor cell

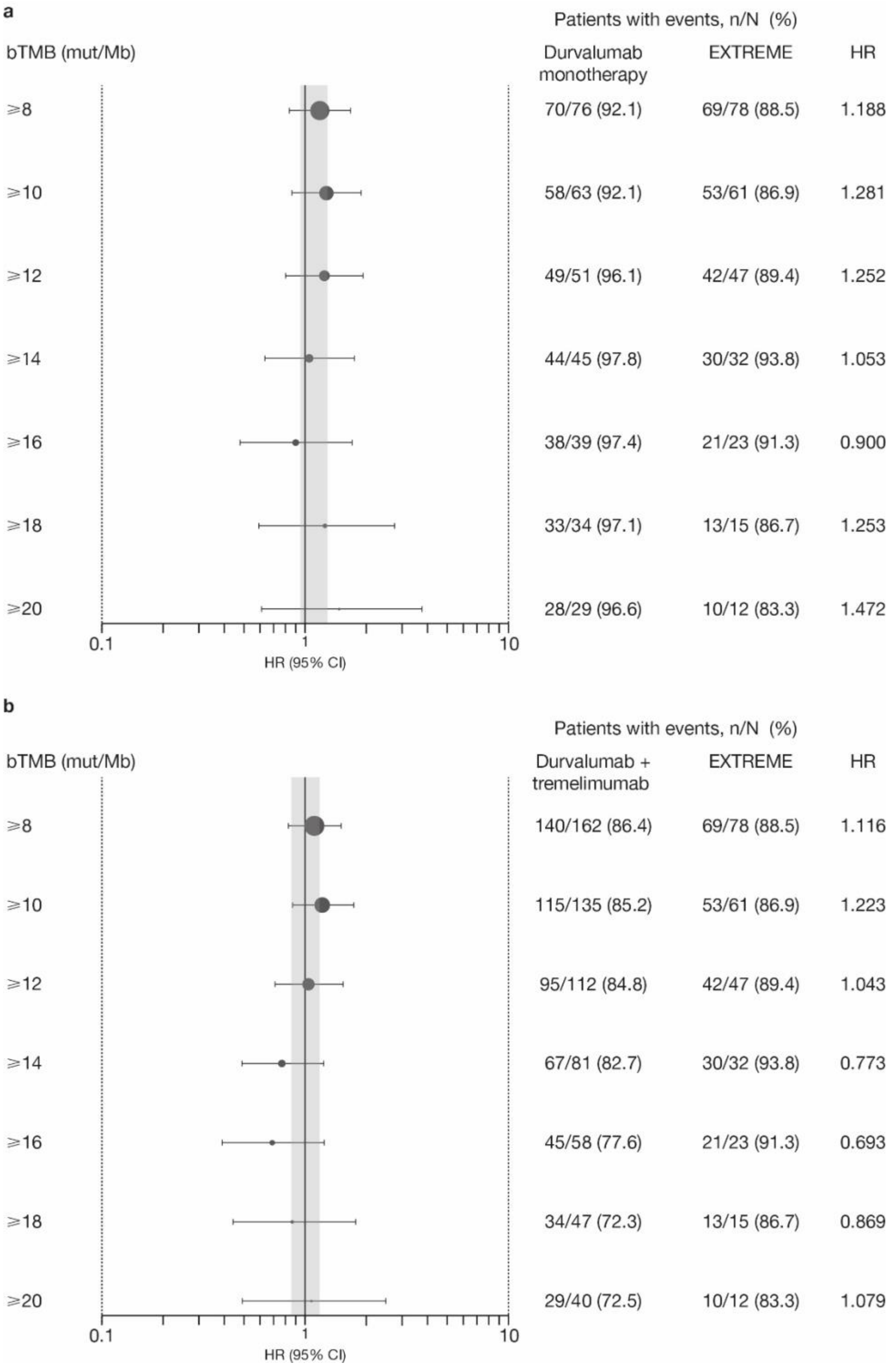

Supplementary Figure 5 OS versus the EXTREME regimen at increasing cut-offs of bTMB for **a**, durvalumab monotherapy and **b**, durvalumab plus tremelimumab

bTMB, blood tumor mutational burden; CI, confidence interval; HR, hazard ratio; mut/Mb, mutations per megabase; OS, overall survival

Population, n (%)	Durvalumab monotherapy (n = 204)	Durvalumab plus tremelimumab (n = 413)	EXTREME (n = 206)	Total (N = 823)
PD-L1 evaluable	203 (99.5)	411 (99.5)	206 (100)	820 (99.6)
PD-L1 TC ≥50%/IC ≥25%	99 (48.8)	190 (46.2)	94 (45.6)	383 (46.7)
PD-L1 TC <50%/IC <25%	104 (51.2)	221 (53.8)	112 (54.4)	437 (53.3)
TC ≥1%	141 (69.5)	291 (70.8)	142 (68.9)	574 (70.0)
TC ≥10%	90 (44.3)	183 (44.5)	84 (40.8)	357 (43.5)
TC ≥25%[a]	63 (30.9)	128 (31.0)	65 (31.6)	256 (31.1)
TC ≥50%	44 (21.7)	73 (17.8)	39 (18.9)	156 (19.0)
IC ≥1%	184 (90.6)	369 (89.8)	188 (91.3)	741 (90.4)
IC ≥10%	174 (85.7)	355 (86.4)	178 (86.4)	707 (86.2)
IC ≥25%	71 (35.0)	151 (36.7)	67 (32.5)	289 (35.2)
IC ≥50%	21 (10.3)	28 (6.8)	10 (4.9)	59 (7.2)
CPS ≥20	76 (37.4)	152 (37.0)	73 (35.4)	301 (36.7)
CPS ≥1	190 (93.6)	380 (92.5)	190 (92.2)	760 (92.7)
PD-L1 unknown	1 (0.5)	2 (0.5)	0 (0)	3 (0.4)

Supplementary Table 1 Prevalence of patients selected by PD-L1 for biomarker analyses

Percentages calculated as a percentage of all evaluable or randomized patients

[a]Prevalence of TC ≥25% was calculated in all randomized patients

Abbreviations: CPS, combined positive score; IC, immune cell; PD-L1, programmed cell death ligand-1; TC, tumor cell

	Durvalumab monotherapy (n = 204)		Durvalumab plus tremelimumab (n = 413)		EXTREME (n = 206)	
	PD-L1 TC ≥50%/ IC ≥25%[a] (n = 99)	PD-L1 TC <50%/ IC <25% (n = 104)	PD-L1 TC ≥50%/ IC ≥25%[a] (n = 190)	PD-L1 TC <50%/ IC <25% (n = 221)	PD-L1 TC ≥50%/ IC ≥25%[a] (n = 94)	PD-L1 TC <50%/ IC <25% (n = 112)
Median OS (95% CI), months	10.9 (9.0–14.3)	9.2 (7.5–11.6)	11.2 (9.5–13.9)	10.2 (8.3–11.7)	10.9 (8.3–13.4)	10.2 (8.0–12.4)
OS HR; *P* value (PD-L1 TC ≥50%/IC ≥25% versus PD-L1 TC <50%/IC <25%)	0.77; 0.138		0.89; 0.345		0.96; 0.804	
12-month OS, % (95% CI)	48.0 (37.8–57.4)	37.2 (27.9–46.5)	49.3 (42.0–56.2)	43.5 (36.8–50.0)	44.0 (33.6–53.8)	43.6 (34.2–52.7)
18-month OS, % (95% CI)	34.7 (25.5–44.1)	28.1 (19.8–37.1)	31.8 (25.3–38.5)	29.6 (23.7–35.8)	30.8 (21.6–40.4)	28.8 (20.6–37.5)
24-month OS, % (95% CI)	27.6 (19.2–36.6)	22.1 (14.6–30.6)	23.9 (18.0–30.1)	21.7 (16.5–27.5)	26.4 (17. 8–35.7)	20.4 (13.4–28.5)

Supplementary Table 2 OS by PD-L1 expression at the TC ≥50%/IC ≥25% cut-off

[a]Data for the PD-L1 TC≥50%/IC≥25% subgroup have been previously reported [1]

Abbreviations: CI, confidence internal; HR, hazard ratio; IC, immune cell; OS, overall survival; PD-L1, programmed cell death ligand-1; TC, tumor cell

	Durvalumab monotherapy			Durvalumab plus tremelimumab			EXTREME		
Population	n	ORR (95% CI), %	CR, %	n	ORR (95% CI), %	CR, %	n	ORR (95% CI), %	CR, %
All-comers [1]	204	17.2 (13.1–24.6)	1.5	413	21.8 (18.7–28.1)	3.9	206	49.0 (43.5–58.6)	1.9
TC ≥1%	141	19.1 (13.0–26.6)	1.4	291	21.3 (16.7–26.5)	4.8	142	52.8 (44.3–61.2)	1.4
TC ≥10%	90	17.8 (10.5–27.3)	0	183	24.6 (18.5–31.5)	6.6	84	53.6 (42.4–64.5)	2.4
TC ≥25%	63	14.3 (6.7–25.4)	0	130	23.8 (16.8–32.1)	5.4	65	52.3 (39.5–64.9)	1.5
TC ≥50%	44	13.6 (5.2–27.4)	0	73	28.8 (18.8–40.6)	5.5	39	46.2 (30.1–62.8)	2.6
IC ≥1%	184	17.9 (12.7–24.3)	1.6	369	21.4 (17.3–26.0)	3.8	188	50.5 (43.2–57.9)	1.6
IC ≥10%	174	17.8 (12.4–24.3)	1.7	355	20.8 (16.7–25.4)	3.4	178	50.6 (43.0–58.1)	1.7
IC ≥25%	71	16.9 (9.0–27.7)	0	151	25.8 (19.1–33.6)	6.0	67	55.2 (42.6–67.4)	3.0
IC ≥50%	21	23.8 (8.2–47.2)	0	28	35.7 (18.6–55.9)	17.9	10	30.0 (6.7–65.2)	0
CPS ≥1%	190	17.9 (12.7–24.1)	1.6	380	21.3 (17.3–25.8)	3.7	190	51.1 (43.7–58.4)	1.6
CPS ≥20%	76	17.1 (9.4–27.5)	0	152	25.0 (18.3–32.7)	5.9	73	53.4 (41.4–65.2)	2.7

Supplementary Table 3 Response rates using different cut-offs for PD-L1 TC, IC, and CPS expression

Abbreviations: CI, confidence interval; CPS, combined positive score; CR, complete response; IC, immune cell; ORR, objective response rate; PD-L1, programmed cell death ligand-1; TC, tumor cell

Parameter[a]	bTMB ≥16 mut/Mb (n = 120)	bTMB <16 mut/Mb (n = 341)	BEP (n = 461)	bTMB unknown (n = 362)
Age (years), median (range)	63.5 (37–79)	60.0 (25–87)	61.0 (25–87)	61.0 (22–89)
Sex, female	23 (19.2)	58 (17.0)	81 (17.6)	53 (14.6)
Race				
White	97 (80.8)	259 (76.0)	356 (77.2)	247 (68.2)
Black or African American	0	2 (0.6)	2 (0.4)	8 (2.2)
Asian	23 (19.2)	79 (23.2)	102 (22.1)	103 (28.5)
Other	0	0	0	4 (1.1)
Missing	0	1 (0.3)	1 (0.2)	0
Nicotine use				
Current	28 (23.3)	79 (23.2)	107 (23.2)	89 (24.6)
Former	73 (60.8)	195 (57.2)	268 (58.1)	218 (60.2)
Never	19 (15.8)	67 (19.6)	86 (18.7)	55 (15.2)
PD-L1 status				
Negative (TC <25%)	73 (60.8)	245 (71.8)	318 (69.0)	249 (68.8)
Positive (TC ≥25%)	47 (39.2)	96 (28.2)	143 (31.0)	113 (31.2)
HPV status (OPC only)				
Positive	18 (15.0)	56 (16.4)	74 (16.1)	50 (13.8)
Negative	23 (19.2)	58 (17.0)	81 (17.6)	68 (18.8)
Missing	0	4 (1.2)	4 (0.9)	5 (1.4)
WHO/ECOG PS				

0	36 (30.0)	137 (40.2)	173 (37.5)	135 (37.3)
1	84 (70.0)	204 (59.8)	288 (62.5)	226 (62.4)
2	0	0	0	1 (0.3)
Primary tumor location				
Oral cavity	20 (16.7)	110 (32.3)	130 (28.2)	118 (32.6)
Oropharynx	41 (34.2)	118 (34.6)	159 (34.5)	123 (34.0)
Hypopharynx	19 (15.8)	49 (14.4)	68 (14.8)	47 (13.0)
Larynx	40 (33.3)	64 (18.8)	104 (22.6)	74 (20.4)
Tumor grade				
1	20 (16.7)	48 (14.1)	68 (14.8)	65 (18.0)
2	50 (41.7)	149 (43.7)	199 (43.2)	146 (40.3)
3	26 (21.7)	71 (20.8)	97 (21.0)	78 (21.5)
4	2 (1.7)	5 (1.5)	7 (1.5)	3 (0.8)
Unassessable	21 (17.5)	64 (18.8)	85 (18.4)	68 (18.8)
Missing	1 (0.8)	4 (1.2)	5 (1.1)	2 (0.6)
AJCC stage				
0	2 (1.7)	0	2 (0.4)	1 (0.3)
I	8 (6.7)	19 (5.6)	27 (5.9)	32 (8.8)
II	8 (6.7)	37 (10.9)	45 (9.8)	31 (8.6)
III	19 (15.8)	63 (18.5)	82 (17.8)	62 (17.1)
IVA	52 (43.3)	142 (41.6)	194 (42.1)	166 (45.9)
IVB	11 (9.2)	23 (6.7)	34 (7.4)	26 (7.2)

IVC	19 (15.8)	54 (15.8)	73 (15.8)	42 (11.6)
Missing	1 (0.8)	3 (0.9)	4 (0.9)	2 (0.6)
Time from diagnosis to first dose				
≤12 months	32 (26.7)	117 (34.3)	149 (32.3)	123 (34.0)
>12 months	88 (73.3)	224 (65.7)	312 (67.7)	222 (61.3)
Missing	0	0	0	17 (4.7)
Overall disease classification				
Recurrent	23 (19.2)	100 (29.3)	123 (26.7)	128 (35.4)
Metastatic	96 (80.0)	237 (69.5)	333 (72.2)	227 (62.7)
Other	1 (0.8)	4 (1.2)	5 (1.1)	7 (1.9)

Supplementary Table 4 Baseline patient characteristics by bTMB status in the BEP and bTMB unknown population

[a]Parameters are n (%) unless otherwise specified

Abbreviations: AJCC, American Joint Committee on Cancer; BEP, bTMB evaluable population; bTMB, blood tumor mutational burden; ECOG, Eastern Cooperative Oncology Group; HPV, human papillomavirus; mut/Mb, mutations per megabase; OPC, oropharyngeal cancer; PD-L1, programmed cell death ligand-1; PS, performance status; TC, tumor cell; WHO, World Health Organization

Population, n (%)	Durvalumab monotherapy (n = 204)	Durvalumab plus tremelimumab (n = 413)	EXTREME (n = 206)	Total (N = 823)
NLR evaluable	204 (100)	413 (100)	206 (100)	823 (100)
NLR ≤7	142 (69.6)	270 (65.4)	144 (69.9)	556 (67.6)
NLR >7	62 (30.4)	143 (34.6)	62 (30.1)	267 (32.4)
NLR unknown	0 (0)	0 (0)	0 (0)	0 (0)

Supplementary Table 5 Prevalence of patients selected by NLR for biomarker analyses

Abbreviations: NLR, neutrophil-to-lymphocyte ratio

NLR level	Parameter	Durvalumab monotherapy	Durvalumab plus tremelimumab	EXTREME
≤4	Number of patients with events,[a] n (%)	68/80 (85.0)	113/138 (81.9)	60/77 (77.9)
	Median OS (95% CI), months	16.5 (11.8–20.1)	15.2 (11.4–18.8)	12.4 (9.1–15.8)
	OS HR[b] (95% CI); *P* value	1.02 (0.71–1.45); 0.835	0.99 (0.72–1.38); 0.819	–
≤8	Number of patients with events,[a] n (%)	134/154 (87.0)	254/305 (83.3)	127/155 (81.9)
	Median OS (95% CI), months	11.6 (9.2–14.6)	13.4 (11.1–15.2)	11.2 (9.4–13.6)
	OS HR[b] (95% CI); *P* value	1.09 (0.85–1.39); 0.632	0.99 (0.80–1.23); 0.923	–

Supplementary Table 6 OS by NLR level

[a]Patients who had not died at the time of analysis were censored at the time they were last known to be alive and are therefore excluded from the number of events

[b]HR and CI were calculated versus the selected patients in the EXTREME arm

Abbreviations: CI, confidence interval; HR, hazard ratio; NLR, neutrophil-to-lymphocyte ratio; OS, overall survival

Reference

1. Psyrri A, Fayette J, Harrington K, et al. Durvalumab with or without tremelimumab versus the EXTREME regimen as first-line treatment for recurrent or metastatic squamous cell carcinoma of the head and neck: KESTREL, a randomized, open-label, phase III study. Ann Oncol. 2023;34:262–74.
